# Clinical Practice Evolvement for Post-Operative Prostate Cancer Radiotherapy—Part 1: Consistent Organs at Risk Management with Advanced Image Guidance

**DOI:** 10.3390/cancers15010016

**Published:** 2022-12-20

**Authors:** Brady S. Laughlin, Stephanie Lo, Carlos E. Vargas, Todd A. DeWees, Charles Van der Walt, Katie Tinnon, Mason Beckett, Dean Hobbis, Steven E. Schild, William W. Wong, Sameer R. Keole, Jean-Claude M. Rwigema, Nathan Y. Yu, Edward Clouser, Yi Rong

**Affiliations:** 1 Department of Radiation Oncology, Mayo Clinic, 5881 E Mayo Blvd., Phoenix, AZ 85054, USA; 2 Department of Qualitative Health Sciences, Section of Biostatistics, Mayo Clinic Arizona, 13400 E Shea Blvd, Scottsdale, AZ 85259, USA

**Keywords:** post prostatectomy radiotherapy, image guidance, CBCT, minimal bladder contour

## Abstract

**Simple Summary:**

Organs at risk (OARs) management (rectum and bladder) is evaluated in patients receiving post-prostatectomy radiation. The role of full bladder instruction and the use of the endorectal balloon is evaluated. The efficacy of this practice was based on daily image and dose delivery using high-quality iterative cone-beam CT (iCBCT). The analysis revealed that a minimal bladder contour can be generated and followed to ensure sufficient bladder sparing. An endorectal balloon is not needed for sufficient target coverage or OAR sparing.

**Abstract:**

Purpose: Post-operative prostate cancer patients are treated with full bladder instruction and the use of an endorectal balloon (ERB). We reassessed the efficacy of this practice based on daily image guidance and dose delivery using high-quality iterative reconstructed cone-beam CT (iCBCT). Methods: Fractional dose delivery was calculated on daily iCBCT for 314 fractions from 14 post-operative prostate patients (8 with and 6 without ERB) treated with volumetric modulated radiotherapy (VMAT). All patients were positioned using novel iCBCT during image guidance. The bladder, rectal wall, femoral heads, and prostate bed clinical tumor volume (CTV) were contoured and verified on daily iCBCT. The dose-volume parameters of the contoured organs at risk (OAR) and CTV coverage were assessed for the clinical impact of daily bladder volume variations and the use of ERB. Minimum bladder volume was studied, and a straightforward bladder instruction was explored for easy clinical adoption. Results: A “minimum bladder” contour, the overlap between the original bladder contour and a 15 mm anterior and superior expansion from prostate bed PTV, was confirmed to be effective in identifying cases that might fail a bladder constraint of V65% <60%. The average difference between the maximum and minimum bladder volumes for each patient was 277.1 mL. The daily bladder volumes varied from 62.4 to 590.7 mL and ranged from 29 to 286% of the corresponding planning bladder volume. The bladder constraint of V65% <60% was met in almost all fractions (98%). CTVs (D90%, D95%, and D98%) remained well-covered regardless of the absolute bladder volume daily variation or the presence of the endorectal balloon. Patients with an endorectal balloon showed smaller variation but a higher average maximum rectal wall dose (D0.03mL: 104.3% of the prescription) compared to patients without (103.3%). Conclusions**:** A “minimum bladder” contour was determined that can be easily generated and followed to ensure sufficient bladder sparing. Further analysis and validation are needed to confirm the utility of the minimal bladder contour. Accurate dose delivery can be achieved for prostate bed target coverage and OAR sparing with or without the use of ERB.

## 1. Introduction

Radiotherapy (RT) to the prostate bed is often recommended in post-prostatectomy settings [1]. Variations in daily bladder and rectum filling can potentially lead to intra- and inter-fractional target motion, thus impacting doses delivered to the clinical target volume (CTV) [2,3]. Intra-fractional errors can also occur given the continuous accumulation of urine in the bladder and bowel movements during treatment [4,5]. To mitigate these variations, patients are routinely instructed to have a full bladder to minimize bowel and bladder dose [5]. Although a full bladder may have dosimetric benefits, it must be obtained consistently in simulation and in subsequent treatments. With the advent of hypofractionation and SBRT in the post-prostatectomy setting, more stringent dosimetric constraints may be required for organs at risk (OARs) [6,7,8]. Thus, an endorectal balloon (ERB) may need to be considered to immobilize the target and to spare normal organs by pushing away the large bowel and inflating the rectal volume [9,10]. While an ERB may lead to more consistent rectal filling and target immobilization, it can lead to patient discomfort and an increased anterior rectal wall dose [10,11]. Further data are needed regarding the dosimetric benefit of margin reduction in OARs [12,13].

Appropriate margins are needed for accurate delivery of RT, accounting for inter- and intra-fractional variation as well as image-guided radiotherapy (IGRT) [2,14,15,16]. Soft tissue contrast on daily cone-beam computed tomography (CBCT) is essential for ensuring accurate daily target positioning. However, traditional CBCT images based on the Feldkamp–Davis–Kress (FDK) filter-back projection algorithm can have poor soft tissue contrast due to scattering contamination [17,18]. Iterative CBCT (iCBCT), a new commercial reconstruction technique, has been evaluated by our group and demonstrated improved image quality for IGRT for various disease sites [19]. With enhanced IGRT, we aim to quantify the benefit of bladder filling and the use of an ERB for post-prostatectomy RT. We hypothesized that target coverage and OAR sparing would not be affected by daily variation if a minimal bladder volume is achieved regardless of the use of an endorectal balloon.

## 2. Materials and Methods

Approval was obtained from the Mayo Clinic Institutional Review Board (IRB #22-001608) prior to performing this retrospective analysis. Fourteen prostate cancer patients treated with salvage RT following prostatectomy for rising PSA were included in this study. A retrospective study on a total of 314 fractions (158 fractions in 8 patients with ERB and 156 in 6 patients without ERB) was performed for post-prostatectomy RT patients treated with volumetric modulated arc therapy (VMAT). At simulation, patients were instructed to lie flat on the simulation tabletop with legs in leg immobilizers and arms on the chest holding a blue ring. Prior to the simulation, all patients were instructed to have a bowel movement. If patients were not able to have a bowel movement, a fleet enema was used. For patients using the ERB, the ERB was coated with lubricant and placed into the rectum by a radiation therapist at simulation. The ERB was then filled with 100 mL of water. All patients were instructed to follow specific bladder filling instructions at subsequent treatments: 45 min prior to treatment check-in, patients may empty their bladder and then immediately drink one bottle (16.9 oz) of water. The bladder filling was confirmed by the simulation therapists on the CT simulation scan. All patients were instructed to have a bowel movement prior to radiation treatments.

The prostate bed CTV was contoured on the planning CT for each patient per the Faculty of Radiation Oncology Genito-Urinary Group (FROGG) consensus guidelines [20]. The apex of the prostate was contoured utilizing an MRI to the plane where the puborectalis muscle is at the level of the urethra. The seminal vesicle bed was not contoured for patients without seminal vesicle invasion on final pathology. If there was seminal vesicle invasion, the involved side was contoured to the distal portion of the vas deferens. The retropubic space was contoured for the initial half of the pubic bone height.

During the treatment of the patients identified for this study, treating therapists were following our old departmental policy for bladder filling level, which is to ensure at least 50% of the planning bladder volume, or have patients drink more water and wait 30 min before re-CBCT until the 50% bladder filling is achieved.

**Figure 1:**
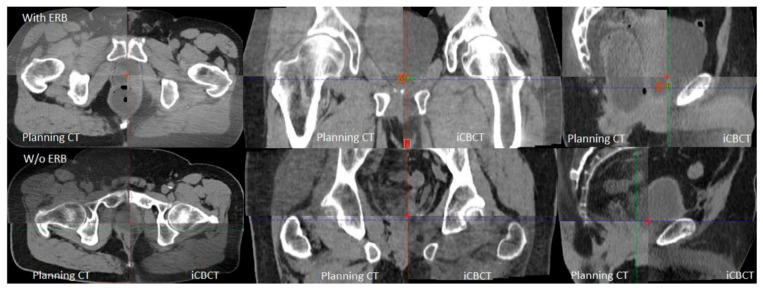
Axial, coronal, and sagittal views demonstrating planning CT and iCBCT image guidance to identify rectal wall and bladder interface and space between bladder and obturator musculature.

**Figure 2:**
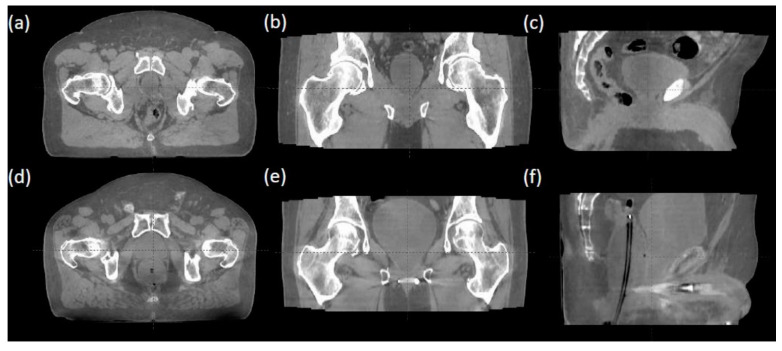
(**a**–**c**) iCBCT reconstruction of a patient without endorectal balloon. (**d**–**f**) iCBCT reconstruction of a patient with endorectal balloon.

Each fraction’s iCBCT was taken prior to treatment for target localization using an On-Board Imager ^®^ (Varian Medical Systems, Palo Alto, CA, USA) with a Pelvis Imaging mode setting (125 kVp, 1080 mAs, 900 projections, and 46.5 cm field of view) and Acuros CTS-based iterative reconstruction algorithm [21,22]. Upon confirming bladder filling by daily iCBCT, therapists then performed an automatic image registration between the planning CT and iCBCT with six degrees of freedom for couch turned on, followed by manual fine-tuning based on the rectum and bladder interfaces in axial, sagittal, and coronal views. Figure 1 provides an example of image registration accuracy using iCBCT compared to the planning CT. The rectal wall and bladder interface and the prostate bed from the obturator internus musculature in patients can be clearly differentiated on the iCBCT with and without an endorectal balloon. This soft tissue contrast quality allows therapists to accurately align the patient without seeing a solid target in a post-prostatectomy setting with clear IGRT matching instructions. Figure 2 demonstrates axial, coronal, and sagittal iCBCT reconstruction of a patient without endorectal balloon (Figure 2a–c) and with endorectal balloon (Figure 2d–f).

Prostate bed CTVs, after being copied on daily iCBCT, were reviewed, translated, or rotated if needed, and approved by a radiation oncologist. The CTVs for two fractions were observed that required deformation and were excluded from the study. Organs at risk, including the bladder, rectum, rectal wall, and femoral heads, were contoured on the planning CT and daily iCBCTs. The 3 mm rectal wall structure was created by extracting the outermost 3 mm from the rectum. The daily dose delivered was calculated during on the daily iCBCT for both CTVs and OARs and compared to the original planned doses. Dose-volume histogram data for the bladder, rectum, and CTV were extracted from the Eclipse treatment planning system (Varian Medical Systems, Palo Alto, CA, USA) and analyzed with MATLAB Version R2021b (MathWorks, Inc., Natick, MA, USA).

Data analysis was performed to identify a minimum threshold of bladder filling that allowed for sufficient bladder sparing, in which most cases met the bladder constraint of V65% <60%. Furthermore, in order to translate this minimal volume into visual guidance that therapists can easily use and make a quick clinical judgment at the time of IGRT, a “minimum bladder” contour was explored.

### Statistical Analysis

Descriptive statistics were calculated for all clinical factors. Repeated-measure mixed models with an auto-regression covariance structure were utilized to model the changes in OAR and/or DVH metric over all treated fractions. As the number of fractions was clinically determined, patients missing data due to different fractionation schedules were considered missing at random and imputation methods were not utilized to reduce potential introduced bias. P-values were presented for all statistical tests, and due to multiple tests for OAR and DVH metrics, we utilized an adjusted α = 0.01; therefore, *p*-values < 0.01 were considered statistically significant. SAS version 9.04.01 was utilized for all statistical analyses.

## 3. Results

All patients previously underwent robotic-assisted prostatectomy prior to post-prostatectomy radiotherapy. Seven (50%) underwent pelvic lymph node dissection. The distribution of the Gleason Grade Group was as follows: four (28.5%) Group 2, three (21.4%) Group 3, three (21.4%) Group 4, and four (28.5%) Group 5. The pathologic AJCC 8th edition staging was: eight (57.1%) T2 and six (42.8%) T3a. Margin status was positive in four (28.5%) patients. Six (42.8%) patients showed evidence of extraprostatic extension. There were no patients with seminal vesicle invasions. All patients underwent salvage radiotherapy. The median PSA prior to radiotherapy was 0.23 (range 0.18–0.52). The median time between surgery and RT start was 4.4 years (0.5–15.7 years). The median AUA score prior to RT was 4 (range: 0–11). The following dose/fractionation schemes were used: 66 Gy in 33 fractions in 10 (71.4%) patients, 52.5 Gy in 20 fractions in 2 (14.3%) patients, and 70.2 Gy in 39 fractions in 2 (14.3%) patients. Ten (71.4%) patients received six months of androgen deprivation therapy (ADT). Radiotherapy was well tolerated with mild side effects: six (42.8%) grade 1 urinary toxicity and three (21.4%) grade 2 toxicity. Three (21.4%) had baseline-adjusted acute grade 2 urinary incontinence. One (7.1%) patient had baseline-adjusted grade 2 late urinary incontinence.

**Figure 3:**
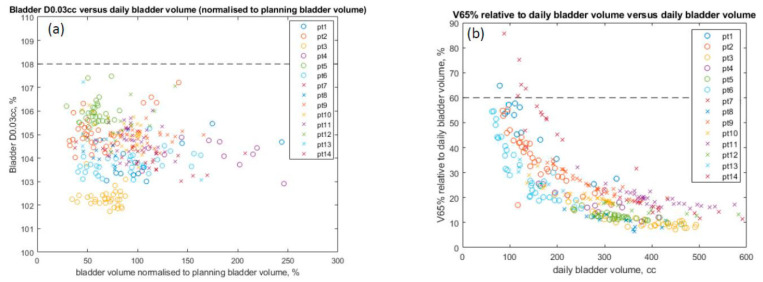
(**a**) Maximum bladder dose (0.03 mL) versus daily bladder volume. Maximum accepted D0.03mL of 108% is indicated with dashed black line. (**b**) V65% relative to daily bladder volume versus daily bladder volume. Constraint of V65% <60% is indicated by the dashed black line. Patients 1–6 (circle) use no endorectal balloons. Patients 7–14 (crosses) use endorectal balloons.

**Figure 4:**
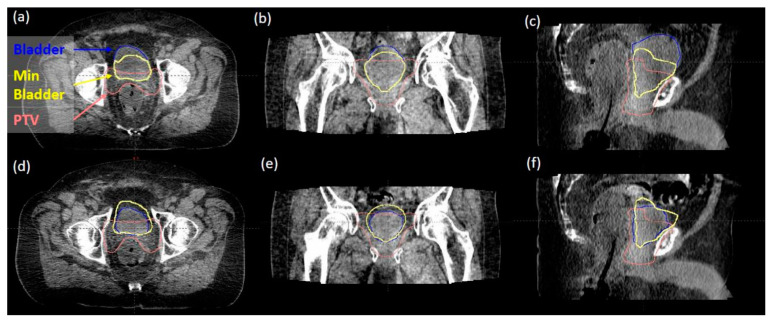
(**a**–**c**) an example of a daily bladder volume that passed the V65% >60% constraint. The bladder (blue) extends anteriorly and superiorly beyond the “minimum bladder” contour (yellow). (**d**–**f**) An example of a daily bladder volume that failed the V65% >60% constraint. The bladder does not completely extend anteriorly and superiorly beyond the “minimum bladder” contour.

In reviewing 314 daily iCBCT images for 14 patients, the initial mean bladder volume at simulation was 319.325 mL (range 113.63–562.66 mL), while the daily bladder volume ranged from 62.4 mL to 590.7 mL over the course of treatment. There was a 29% to 286% variation observed from daily CBCTs compared to the initial bladder volume at simulation. The maximum dose to the bladder D0.03mL <103–108% with large variations in daily bladder volume is reported in Figure 3a. All cases were below our institutional max bladder dose of 108%, with no differences seen in patients with or without ERB. The bladder dose constraint V65% <60% (with respect to daily bladder volume) was met in 309 of 314 fractions (Figure 3b). A “minimum bladder” contour was created by expanding the PTV in the superior and anterior directions by 15 mm and then intersecting this volume with the planning bladder volume. The value of 15 mm was determined based on the consensus of our genitourinary (GU) expert physicians, as well as an estimate of the photon dose fall-off distance to below 20% of the prescription dose. This “minimum bladder” structure was validated based on what visually separated the daily bladder volumes that failed the V65% <60% constraint from the bladder volumes that passed. Figure 4 demonstrates examples of how the minimal bladder contour differentiates between passing (Figure 4a–c) and failing the V65 <60% constraint (Figure 4d–f). The daily bladder volumes in the five failing fractions all failed to completely extend anteriorly and superiorly beyond this “minimum bladder” contour. Figure 1b further shows that the V65 of the bladder decreases as the daily volume of the bladder increases.

**Figure 5:**
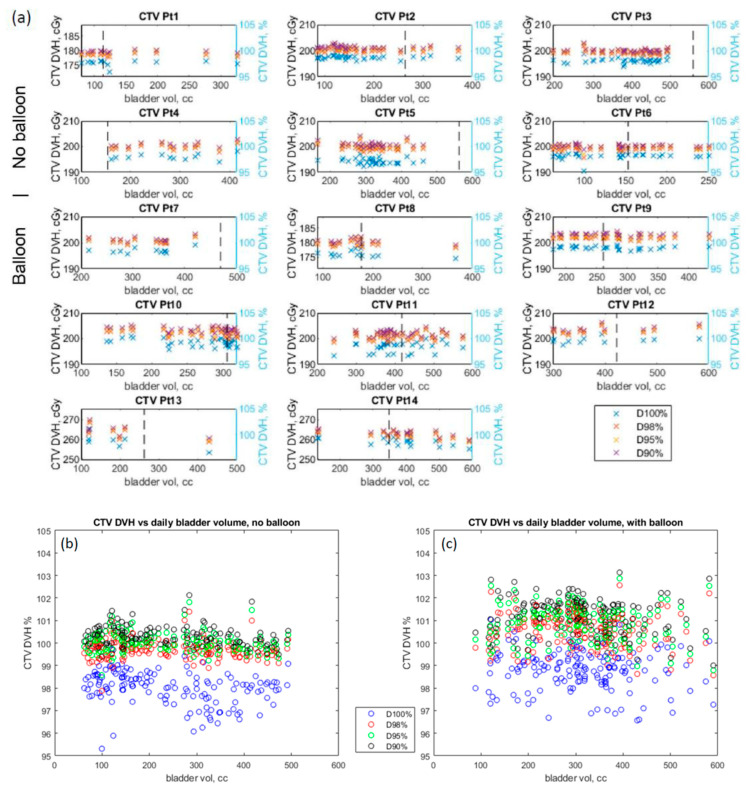
(**a**) CTV coverage for each patient as a function of daily bladder volume. The dashed black line indicates the planning bladder volume. CTV coverage as a function of daily bladder volume for patients without endorectal balloon (**b**) and with endorectal balloon (**c**).

**Figure 6:**
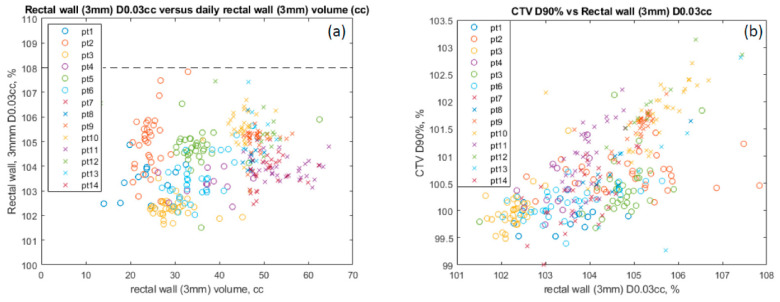
(**a**) Maximum rectal wall (3 mm) dose. (**b**) CTV D90% coverage vs. max rectal wall dose. Patients 1–6 (circle) use no endorectal balloons. Patients 7–14 (crosses) use endorectal balloons.

Table 1 provides statistical analysis showing that the median change in the bladder V65 was −0.00084% (*p* < 0.0001). With the ERB, the rectal V45 is higher by 10.3 cGy (*p* < 0.001). The daily change in bladder volume resulted in a median change in the CTV D90% of 0.0037 cGy (*p* = 0.006) and in the CTV D95% of −0.0017 cGy (*p* = 0.027), which is clinically not significant. Figure 5a demonstrates consistent CTV coverage (D90%, D95%, and D98%) by patient, despite the large bladder volume variation and with (Pt 7–14) or without (Pt 1–6) an ERB. The dashed vertical line in each figure shows the initial bladder volume at simulation. CTVs (D90%, D95%, and D98%) stayed consistently covered with a range of 95% to 103% for all patients over all the fractions regardless of the absolute bladder volume daily variation, no balloon (Figure 5b) or with an endorectal balloon (Figure 4c). Patients with an ERB had a median dose increase of 1.61 cGy and 1.54 cGy in CTV D90% and D95% (*p* < 0.001), respectively. Patients with balloons (crosses) had a smaller variation but a higher average maximum rectal wall dose (D0.03mL: 104.3% of the prescription) compared to patients without (103.3%) (circles) (Figure 6a). All rectal wall maximum doses (D0.03mL) met the dose constraints (<108% of the prescription) regardless of the rectal wall volume, or the use of ERBs. Figure 6b shows the CTV D90% coverage with respect to the maximum wall doses, with or without an ERB, also indicating higher rectal wall doses with ERB (crosses).

## 4. Discussion

To our knowledge, this is one of the few institutional series evaluating the use of a minimum bladder contour with daily dose calculation by high-quality iCBCT scans for patients receiving post-prostatectomy RT. Although a full bladder may have advantages, such as pushing the small bowel out of the field, it can be challenging for patients to maintain and difficult for therapists to decide when to proceed with treatment. In this study, we studied the need for updating the traditional practice guideline of having a full bladder and the use of ERBs for post-prostatectomy patients. There have been multiple studies evaluating whether changes in bladder or rectum volume impact dose delivery to the prostate bed in patients receiving post-prostatectomy radiation [23,24,25,26]. In a study of nine patients receiving post-prostatectomy radiation, Fiorino et al. demonstrated that bladder volumes reduce in size over the course of treatment and the superior half of the rectum shifted anteriorly in six of nine patients, which correlated to a corresponding shift in the posterior border of the defined CTV [2]. Bell et al. assessed 377 CBCT of 40 patients who received post-prostatectomy radiation and found that the change in bladder diameter resulted in potential geographic miss of dose delivery in the upper prostate bed [3]. In a study of 10 patients undergoing post-RP IGRT, Showalter et al. also demonstrated posterior bladder wall variability. The conclusions reached in these studies are based on traditional FDK CBCT, with known low soft tissue contrast and a high level of image artifacts. Contrary to these studies, our study demonstrated little impact on CTV with the changes in bladder volume if it was above a certain minimum threshold when novel daily iCBCTs were utilized.

The investigation of a minimal bladder contour to help guide daily patient setup and reproducibility for prostate bed radiotherapy patients has not drawn much attention. Only two relevant studies were found in literature. Happersett et al. investigated the concept of a minimal bladder contour in 64 patients with intact prostate cancer receiving SBRT who had delays in treatment due to the underfilling of the bladder in 35/115 fractions [27]. In their study, a minimal bladder contour was determined by deforming the bladder contour at simulation with decreasing margins and identifying the contour that met bladder constraints (maximum <105%, V20Gy <50%, and V 36Gy <10%) [27]. With the use of a minimal bladder contour, CBCT predicted that the bladder would be large enough for 22/35 fractions (63%). The same group applied the use of a minimal bladder contour in 20 patients receiving 5-fraction SBRT [28]. Out of 100 fractions, there was only 1 fraction where bladder constraints were exceeded, but this was because the approved CBCT bladder volume was smaller than the minimal bladder contour. Additionally, the group demonstrated a reduction in the average treatment time compared to historical treatment times (26 ± 15 min/fraction vs. 31.5 ± 20 min/fraction) [28]. In the present study, we demonstrated that a minimal bladder volume is sufficient to adequately maintain target coverage and meet bladder dose constraints. Our method of producing a minimal bladder contour with 15 mm expansion anteriorly and superiorly from the PTV, overlapping with the original bladder contour, has several advantages, including being straightforward for dosimetrists to create and not requiring deformation, as well as being easy for therapists to visually check against to determine sufficient bladder filling. The minimum bladder size that Happersett and colleagues determined was 157 mL, which is consistent with the threshold seen in our study. We demonstrated a consistent finding that a minimum bladder volume can be used to ensure accurate delivery. Therefore, there is no need for a daily full bladder requirement.

Cone-beam CTs are associated with poor image quality due to the degradation caused by scattering artifacts [17,18]. The degradation in image quality makes it difficult to delineate the interface between the anterior rectal wall and the bladder. With the advancement in image guidance and more conformal radiation techniques, the omission of ERBs has become the standard of care for intact prostate and prostate bed radiotherapy receiving standard fractionation [29,30]. Additionally, patients are instructed to have a bowel movement prior to radiation treatment. Previous data have demonstrated that a distended rectum at simulation resulted in reduced toxicity but a much higher recurrence rate [31]. As the field may be moving towards hypo-fractionation, smaller target margins may be utilized. The requirements for internal organ immobilization and imaging alignment are more stringent in consideration of higher fractional doses and fewer fractions to compensate for organ motion or misalignment. Therefore, the use of an ERB may be reconsidered for organ management and better target immobilization. In the meantime, improved image quality in iCBCT provides better soft tissue contrast and Hounsfield unit uniformity [32,33]. Figure 1 and Figure 2 show examples of iCBCT for patients without and with ERB, respectively. With the clear identification of the rectal wall and bladder interface, accurate alignment can be achieved in all three views allowing the omission of daily ERB placement. The present study revisiting the use of ERBs with an improved CBCT image quality and organ consistency (i.e., bladder and rectum) is justified.

The daily CTV dose coverage evaluation in this study was achieved by copying CTV from the planning CT to the daily iCBCT while only allowing translational or rotational corrections, assuming that there was no CTV volume deformation throughout the treatment. In their phase II trial evaluating post-prostatectomy stereotactic body RT, Yoon et al. reported on a volumetric and dosimetric analysis by kilo-voltage CBCT of 18 patients [34]. Volumetric and dosimetric changes were minor due to inter-fraction motion and rotation [34]. A CTV V95% was greater than 93% for 13/18 patients. In patients receiving post-prostatectomy SBRT in a single institution phase II trial, Cao et al. use MRI-guided adaptive RT to account for variations in CTV and OARs [35]. They reported the stability of CTV volume and shape with a 3.0% median volume change. MRI-guided adaptive therapy was deemed to be beneficial in approximately 78.2% of the 55 fractions due to target under-coverage, exceeding OAR constraints, or both [35]. In their initial report of a phase III trial evaluating MRI image-guided SBRT versus CT-guided SBRT for intact prostate cancer, Kishan et al. demonstrated that acute grade >2 GU toxicity was reduced in patients who received MRI-guided SBRT [36]. However, the reduction in acute grade 2 GU toxicity might be due to the reduced size of the planning target volume expansion: the planning target margin was 4 mm and 2 mm for the CT arm and MRI arm, respectively [36]. Based on the prostate bed CTV studies mentioned previously, it was felt to be appropriate to proceed with no CTV deformation in the daily iCBCT. Additionally, little deformation may only be accounted for by the presence of intra-observer variation or prostate bed target definition.

Rectal filling has previously been demonstrated in MRI studies assessing intra-fraction motion to be predictive of prostate motion [15,37,38]. ERBs are used to immobilize the prostate and expand the rectal volume [10]. A comparative study of 7 of 14 patients treated with ERBs demonstrated a reduction in target volume and rectum motion [39]. The benefit of ERB in terms of rectal dose sparing has been controversial, or even misleading, in the literature. Smeenk et al. demonstrated 8% and 5% reductions in rectal V30 and V40 in prostate bed patients with ERBs. However, the reduction is likely to have resulted from the reduced CTV volume with an ERB (110 ± 20 mL), compared to the one without an ERB (117 ± 27 mL) [12]. On the other hand, Jameson et al. demonstrated that the use of endorectal balloons did not lead to any significant improvements in PTV coverage or OAR sparing [13]. The presence of an endorectal balloon provides a clear outline visible on daily CBCT, allowing for positional accuracy. Our study demonstrated a clinically insignificant difference in daily CTV D90% (1.61 cGy) with the use of an endorectal balloon as well as a significantly higher maximum anterior rectal wall dose.

This study has several strengths and limitations. With a thorough evaluation of 314 daily fractions of iCBCT and their corresponding fractional dose, we validated the effectiveness of the use of a “minimum bladder” instruction and demonstrated the lack of clinical benefit of using ERBs for prostate bed patients using novel iCBCT for daily IGRT. A limitation of the study is that it only corresponded to 14 patients. However, due to the similar anatomy of prostate bed patients and consistent planning techniques, our conclusion is valid with the study of 314 daily fractions. Male pelvic anatomy is very similar in general, although the biggest variations are in the bladder and rectum. With more than 300 datapoints from daily iterative cone-beam CT, we accounted for general variations in bladder or rectum volume, regardless of whether they belong to the same patient or different patients. Nonetheless, we express caution regarding the applicability and generalizability of our findings given this was carried out in 14 patients. Within our study, the CTV was copied over to the daily iCBCT without much deformation seen. An expert radiation oncologist determined whether the CTV was within the correct location, with only two fractions having deformations and subsequently being excluded. Because there was previously support for minimum deformations with MR-guided RT by Kishan et al., this was felt to be the best way for us to proceed in our study. As CTVs were copied over with confirmation of no deformations by a radiation oncologist, this introduces a limitation to the study, since the conclusion applies to those cases with minimal CTV deformation during treatment. The study could be enhanced if more patient datasets were included to see a larger variation and deformation in CTV. It is possible that there was an underestimation of the extent of movement or contour variation. While the minimal bladder volume may provide a guide for therapists, its reliability in the daily clinical setting has yet to be tested. Another limitation is the inability to correlate bladder filling instruction and the use of endorectal balloons with quality of life during treatment and toxicity. Further analysis is needed to determine whether the minimal bladder contour or the use of ERBs, while accounting for variation in CTV, impacts the ability to meet bladder and rectum dose-volume constraints. A future study will evaluate the use of minimal bladder filling contour and patients’ treatment experience.

## 5. Conclusions

With the use of daily iCBCT, we determined that complete bladder filling and the use of endorectal balloons are not necessary. We theorize that a minimal bladder contour may be created as a guide for bladder filling, without compromising the target coverage of increased doses to OARs. Further analysis is needed to validate the minimal bladder contour and its applicability to a wider population of patients undergoing post-prostatectomy radiation. The omission of ERBs for prostate bed patients reduces patient discomfort and the maximum dose to the rectal wall, and increases the clinical workflow efficiency without sacrificing target coverage or tissue sparing.

## Figures and Tables

**Table 1:** Impact of Bladder Filling Change and Rectal Balloon on Target Coverage and Organs at Risk.

Dose Constraint	Median Change	*p*-Value
Daily Change in Bladder Volume		
CTV D90%	0.0037 CGy	*p* = 0.006
CTV D95%	−0.0017 cGy	*p* = 0.027
Bladder V65%	−0.00084%	*p* < 0.001
Rectal Balloon		
CTV D90%	1.61 cGy	*p* < 0.001
CTV D95%	1.54 cGy	*p* < 0.001
Rectum V45%	10.3 cGy	*p* < 0.001

## Data Availability

Research data are stored in an institutional repository and will be shared upon request to the corresponding author.

## Abbreviations

CBCT	cone-beam computed tomography
CTV	Clinical target volume
DVH	dose volume histogram
ERB	Endorectal balloon
FDK	Feldkamp–Davis–Kress
FROGG	Faculty of Radiation Oncology Genito-Urinary Group
GU	genitourinary
iCBCT	iterative cone-beam computed tomography
IGRT	Image-guided radiotherapy
MRI	Magnetic resonance imaging
OAR	organ-at-risk
PTV	planning target volume
RT	Radiotherapy
VMAT	Volumetric modulated radiation therapy
